# Temporal changes in characteristics and external validity of randomized controlled trials in older people from 2012 to 2019

**DOI:** 10.1186/s12877-023-04018-6

**Published:** 2023-05-24

**Authors:** Estelle van Eijk, Yanna M. van der Spek, Floor J. A. van Deudekom, Frederiek van den Bos, Simon P. Mooijaart, Stella Trompet

**Affiliations:** 1 Section of Gerontology and Geriatrics, Department of Internal Medicine, Leiden University Medical Center, PO Box 9600, 2300 RC Leiden, the Netherlands; 2 Department of Geriatrics, OLVG, Amsterdam, The Netherlands; 3 LUMC Center for Medicine for Older People, Leiden, The Netherlands

**Keywords:** Geriatric assessments, Temporal changes, Older individuals, Systematic review, Randomized controlled trials

## Abstract

**Background:**

Older individuals are often underrepresented in clinical trials. In 2012 only 7% of RCT’s specifically studied older people and their geriatric characteristics were poorly reported. The aim of this review was to investigate temporal changes in characteristics and external validity of randomized controlled trials in older people from 2012 to 2019.

**Methods:**

PubMed was searched for randomized clinical trials (RCTs) published in 2019. Firstly, the proportion of RCTs specially designed for older people were determined by the following criteria: a reported mean age of ≥ 70 years or a lower age cutoff of ≥ 55. Secondly, the trials with a majority of older people, defined by a reported mean age of ≥ 60 years, were screened for reporting of geriatric assessments. Both parts were compared with identical reviews performed in 2012.

**Results:**

From a 10% random sample, 1446 RCTs were included in this systematic review. First, 8% of trials were specifically designed for older people in 2019 compared to 7% in 2012. Secondly, 25% of the trials included a majority of older people in 2019, compared to 22% in 2012. Thirdly, in 52% of these trials in 2019 one or more of the geriatric assessments were reported compared to 34% in 2012.

**Conclusions:**

Although in 2019 the proportion of published RCTs specifically designed for older people remains low, more characteristics on geriatric assessments were reported compared to 2012. Continued efforts should be paid to increase both the number and the validity of trials for older people.

**Supplementary Information:**

The online version contains supplementary material available at 10.1186/s12877-023-04018-6.

## Background

Evidence-based medicine lays the foundation for medical practice, but although the population is ageing, older people are underrepresented in randomized clinical trials (RCTs) [1]. RCTs are considered a high level of evidence, but in 2012 our group found that only 7% of all RCTs published in 2012 were specifically designed for older people [2].

Not only the relatively low mean age in RCTs, but also the lack of information about geriatric domains makes it difficult to apply scientific results to clinical practice [3]. The older population is a very heterogenous patient group regarding the geriatric domains, which include somatic status, physical and mental functioning, social environment and frailty. These four domains can be evaluated using geriatric assessments, which is a multidimensional and multidisciplinary assessment tool to evaluate an older person’s somatic, functional and cognitive status [4]. Geriatric assessments also have prognostic value in several diseases [5] and are therefore advised to take into account to optimize an older person’s treatment decision [6]. To critically assess to what extent the outcomes of RCTs are generalisable to the older patient, a clear description of the geriatric characteristics of a study population is important for a clinician. Van Deudekom et al. investigated this external validity of the same set of RCTs from the year 2012 and found that only 34% of trials including an older study population reported on these geriatric assessments [3].

It is unclear how the representation of older people in RCT’s has developed over the last years and whether the external validity of these trials has improved. Therefore, the aim of the present study was to evaluate temporal changes in characteristics and external validity of randomized controlled trials in older people from 2012 to 2019. To enable comparison over time we used the identical methodology as for the analyses in 2012.

## Methods

The methods of this systematic review are identical to the reviews of Broekhuizen et al. and Van Deudekom et al. [2, 3]. The first review investigated the percentage of RCTs published in 2012 that weres specifically designed for older people, whereas the second review searched the same set of RCTs for trials including an older study population and evaluated the percentage of trials reporting on geriatric assessments. The authors of both articles allowed us to use their methods and data for this review. Since we will replicate the methods of two previously published reviews, this review will consist of two parts but will make use of one article selection.

### Search strategies

A systematic search was conducted (April 1^st^ 2020) to identify RCTs that were published in 2019, this was five years after publication of the two previously executed reviews. The following search strategy was used in the PubMed database:

- ("Randomized Controlled Trial"[Publication Type] OR rct[ti] OR randomized[ti] OR randomised[ti]) AND ("2019/01/01"[PDAT]: "2020/01/01"[PDAT]).

### Study selection

With the use of *IBM SPSS statistics,* a random 10% sample was taken from the search strategy results. This sample was divided over two researchers (EE, YS), who reviewed these publications for in- or exclusion. Articles that were written in English and reported a randomized controlled trial were included. Study protocols, pilot studies, secondary analyses of primary RCTs, systematic reviews and meta-analysis were excluded. Non-human studies and studies describing non-medical interventions were excluded as well. In the first screening round, articles were in- or excluded based on titles and abstracts. Secondly, the remaining full text articles were screened for the similar in- and exclusion criteria. Each publication was reviewed by one reviewer. In case of doubt or uncertainty, a second reviewer was asked for evaluation. When no consensus was reached a third reviewer (ST) made the final decision.

### Data extraction

For all selected RCTs, data was extracted from full text articles by two researchers (EE, YS). The following data was extracted: study characteristics (title and authors), number of participants, mean age, lower and higher age cutoff, study population and primary outcome. For the first 50 articles, data extraction was performed by both reviewers. Since the reproducibility of this data extraction was high, we decided to continue with data extraction per article by only 1 reviewer. If one of the reviewers experienced any difficulty or uncertainty, double reviewing was performed.

Based on study populations and primary outcomes, one reviewer (EE) classified all included RCTs in disease categories according to the International Classification of Diseases (ICD-10) of the World Health Organization (WHO) [7]. Again, double reviewing was performed in case of uncertainty. When no consensus was reached, a third reviewer (ST) made the final decision.

### Age classification

For the first part of this review, the number of RCTs specifically designed for older patients was determined. This was performed identically to the review of Broekhuizen et al. [2]. Criteria for articles to be specifically designed for older people were a reported mean age of 70 years and older or a lower age cutoff for inclusion of 55 years and older.

For the second part of the review, the number of RCTs including a majority of older people was defined. Trials with a reported mean age of 60 years and older were considered to include a majority of older people, similar to the methods of Van Deudekom et al. [3]. These trials were included for further analysis of geriatric assessments. As sensitivity analyses we also changed the age threshold to a mean age of 70 years and older and 80 years and older.

### Geriatric assessments

In the second part of this review, patient characteristics of trials including a majority of older people were screened for reporting of geriatric assessments. This included analysis of both population descriptives and in- and exclusion criteria for the patient characteristics in geriatric assessment. The following geriatric assessments were considered: somatic status, physical and mental functioning, social environment, and frailty. Somatic status included comorbidity scales and polypharmacy scores. Physical functioning consisted of functional performance, mobility and physical capacity. The mental function assessment comprised cognitive status, dementia and the presence of depression or other mood disorders. Description of living situation, marital status, home care or help were included in social environment. Frailty indices or instruments representing the frailty domain were also included.

Geriatric assessment analysis of the first half of the included RCTs for older people was performed separately by two researchers (EE, YS). The results of both reviewers were compared and no disagreements in geriatric assessment analysis were found. Therefore, the second half of the trials was reviewed by one researcher (YS). In case of any uncertainty, consensus was reached after discussion with the second researcher (EE).

To further investigate the external validity of the RCTs including a majority of older people, the primary outcomes of these trials were extracted from the articles and searched for geriatric endpoints. Primary outcomes that fell under the geriatric domains as described above were considered to be geriatric endpoints. The following outcomes were included: outcomes related to mental functioning (i.e. mood, depression, delirium), outcomes related to physical functioning (i.e. walking speed, gait, strength, balance, falls), cognitive function and frailty.

### Statistical analysis

For continuous variables from the trials, measures of central tendency were reported as median with interquartile range (IQR). Dichotomous variables were reported as frequencies and expressed as percentages. The percentage of trials in ICD-10 disease categories were compared between trials specifically designed for older people and trials not specifically designed for older people using Pearson’s Chi Square Test. All analyses were performed with IBM SPSS Statistics version 27.

## Results

**Fig. 1:**
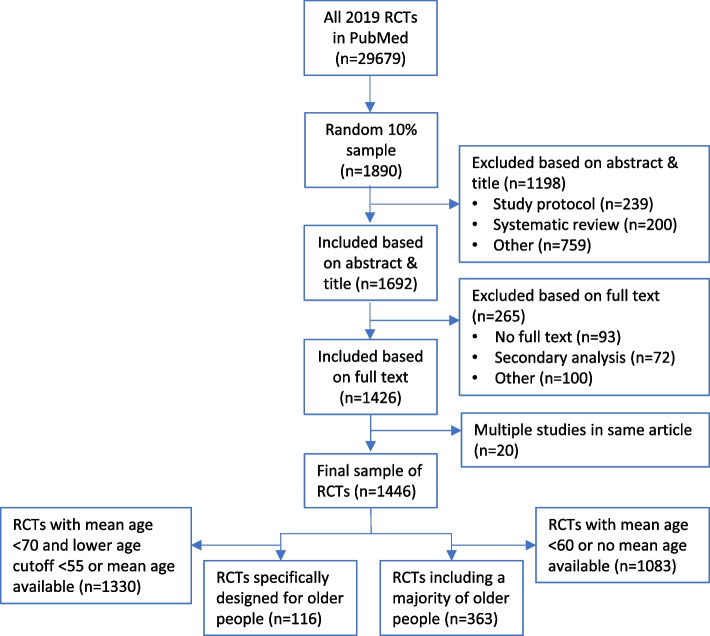
Flow chart of RCT inclusion. Flow chart of the result of the performed search strategy and process of selecting trials specifically designed for older people and trials including a majority of older people. Abbreviations: RCT, randomized controlled trial

The result of the search strategy is depicted in the flowchart in Fig. 1. The PubMed search strategy yielded 29,679 publications. A random 10% sample of these publications was taken, resulting in 2890 selected publications. After screening of abstract and titles, 1692 publications were included. Most excluded publications contained a study protocol (*n* = 239) or described a systematic review instead of a RCT (*n* = 200). For 93 articles, no full text version could be retrieved. After screening of 1692 full text articles, another 172 publications were excluded. This resulted in 1426 included publications. Some of these publications described more than one RCT, leading to a final sample of 1446 RCTs.

### Part 1: RCTs specifically designed for older patients

**Fig. 2:**
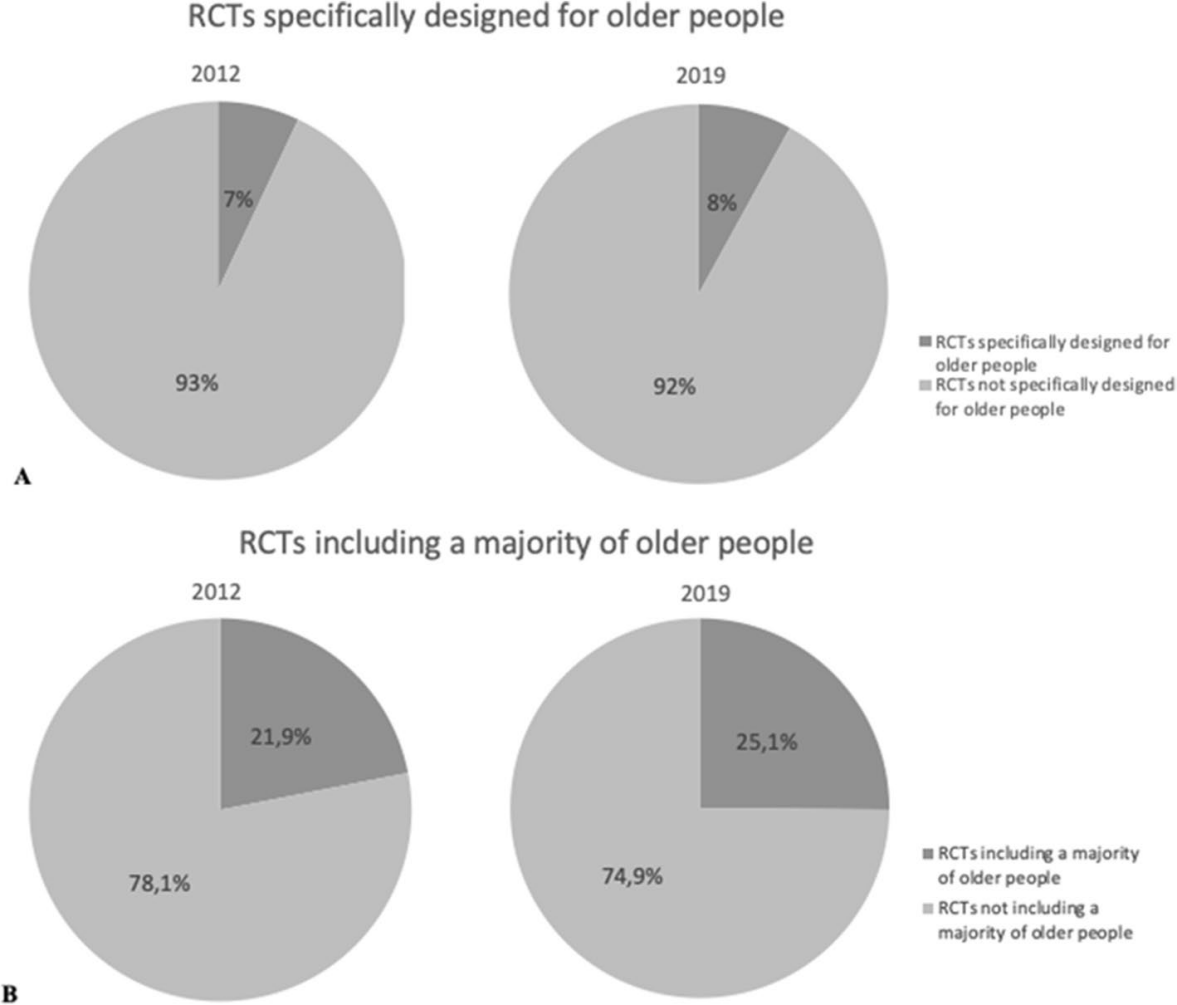
Proportion of RCTs specifically designed for older people in 2012 and 2019 (**A**) and RCTs including a majority of older people in 2012 and 2019 (**B**). Pie chart showing the proportion of trials specifically designed for older people and trials including a majority of older people. A: Trials were classified as specifically designed for older people if minimum trial participant age ≥ 55 years, or if mean trial participant age ≥ 70 years. B: Trials were classified as including a majority of older people if mean trial participant age ≥ 60 years. Abbreviations: RCT, randomized controlled trial

Of the 1446 included RCT’s, 116 of 1446 (8%) trials were specifically designed for older people in 2019 compared to 96 of 1369 (7%) trials in 2012 (Fig. 2A).

Table 1 shows the main trial characteristics of all included trials from the years 2019 compared with 2012. Median participant age in the total sample of all ages in 2019 was 46.5 years (IQR 28.5–60.5) compared to 46 (IQR 29–60) in 2012. Age-related lower cutoff values were applied in 990 of 1446 (68.5%) studies published in 2019 compared to 787 of 1369 (58%) in 2012. Median lower age cutoff value was 18 (IQR 18–20) in both years. The median number of study participants was 84 (IQR 48–142) in 2019 compared to 82 (IQR 40–215) in 2012. In the subset of trials specifically designed for older people, median age was 72 years (IQR 70–74.5) in 2019 compared to 73 years (IQR 71–77) in 2012. Median lower age cut-off was age 60 (IQR 50–65) in 2019 compared to age 65 (IQR49-65) in 2012. More participants are included in trials designed for older adults (median 104; IQR 50–241) compared to trials not designed for older patients (median 84; IQR 48–182) in 2019.

**Table 1:** Main trial characteristics of all included RCTs and trials specifically designed for older people

	Main trial characteristics			
	All included trials		Trials specifically designed for older people	
	2012 *n* = 1369	2019 *N* = 1446	2012 *N* = 96	2019 *N* = 116
Number of participants N, (median, IQR)	82 (40–215	84 (48–182)	125 (48–316)	104 (50–241)
Age of participants, years (median, IQR)	46 (29–60)	46.5 (28.5–60.5)	73 (71–77)	72 (70–74.5)
Lower cut-off value for age, years (median, IQR)	18 (18–20)	18 (18–20)	65 (49–65)	60 (50–65)

Trials were classified as specifically designed for older people if minimum trial participant age ≥ 55 years, or if mean trial participant age ≥ 70 years

Medians and interquartile ranges (difference between 25 and 75^th^ percentile) are reported

*Abbreviations*: *RCT* Randomized controlled trial, *IQR* Interquartile range

In 2019, most trials were classified into WHO disease categories musculoskeletal (*N* = 155; 10.7%), neoplasms (*N* = 142; 9.8%), metabolic (*N* = 135; 9.3%), circulatory (*N* = 121; 8.4%) and mental/behavioural (*N* = 112; 7.7%) (Table 2). In 2012, most trials were categorized into disease categories behavioural, circulatory, metabolic, neoplasms and musculoskeletal. More RCTs specifically designed for older people were categorized in ICD-10 disease categories mental/behavioral (12.1% vs 7.4%), circulatory (15.5% vs 7.7%) and musculoskeletal (19.0% vs 10.0%), compared to RCTs not designed for older patients. The number of trials in disease categories was significantly different in trials specifically designed for older people and trials not specifically designed for older people (*p* = 0.01, Pearson’s Chi square Test).

**Table 2:** Disease categories of 1446 included RCTs in 2019

	All included trials (*N* = 1446)	Trials specifically designed for older people (*N* = 116)	Trials not specifically designed for older people (*N* = 1330)
Infectious	59 (4.1%)	2 (1.7%)	54 (4.3%)
Neoplasms	142 (9.8%)	10 (8.6%)	132 (9.9%)
Blood	18 (1.2%)	1 (0.9%)	17 (1.3%)
Metabolic	135 (9.3%)	5 (4.3%)	130 (9.8%)
Mental/behavioral	112 (7.7%)	14 (12.1%)	98 (7.4%)
Nervous system	78 (5.4%)	3 (2.6%)	75 (5.6%0
Eye	24 (1.7%)	3 (2.6%)	21 (1.6%)
Ear	3 (0.2%)	0 (0%)	3 (0.2%)
Circulatory	121 (8.4%)	18 (15.5%)	103 (7.7%)
Respiratory	57 (3.9%)	3 (2.6%)	54 (4.1%)
Digestive	63 (4.4%)	4 (3.4%)	59 (4.4%)
Skin	33 (2.3%)	2 (1.7%)	31 (2.3%)
Musculoskeletal	155 (10.7%)	22 (19.0%)	133 (10.0%)
Genitourinary	88 (6.1%)	6 (5.2%)	82 (6.2%)
Pregnancy	46 (3.2%)	0 (0%)	46 (3.5%)
Perinatal	34 (2.4%)	0 (0%)	34 (2.6%)
Congenital	9 (0.6%)	0 (0%)	9 (0.7%)
Symptoms	8 0.6%)	0 (0%)	8 (0.6%)
Injury	6 (0.4%)	0 (0%)	6 (0.5%)
External	10 (0.7%)	0 (0%)	10 (0.8%)
Contact with health services	18 (1.2%)	2 (1.7%)	16 (1.2%)
Other	227 (15.7%)	21 (18.1%)	206 (15.5%)

Pearson’s Chi square test: *p* = 0.01

Trials were classified as specifically designed for older people if minimum trial participant age ≥ 55 years, or if mean trial participant age ≥ 70 years

Percentages are reported

*Abbreviations*: *RCT* Randomized controlled trial

### Part 2: external validity of randomized controlled trials including a majority of older people

Trials including a study population with a mean or median participant age of 60 years and older were assumed to be including a majority of older people. 363 of 1446 (25.1%) included 2019 RCTs included a majority of older people, compared to 300 of 1369 (21.9%) trials in 2012 (Fig. 2B).

#### Main trial characteristics of RCTs including a majority of older people

Table 3 shows the comparison of the main trial characteristics of all analyzed articles including a majority of older people from 2019 and 2012. Median participant age was 66 (IQR 63–70) in both 2019 and 2012. The median number of participants per trial was 104 (58–240) in 2019, compared to 114 (IQR 47–288) in 2012. In both years, WHO disease categories circulatory, neoplasms and musculoskeletal were the 3 most frequent trial classifications.

**Table 3:** Main trial characteristics of RCTs including majority of older people

	2012		2019
	(N = 300)		(N = 363)
Number of participants, N (median IQR)	114 (47–288)		104 (58–240)
Age of participants, years (median, IQR)	66 (63–70)		66 (63–70)
Disease categories, N (%)			
Circulatory	74 (25)	Circulatory	75 (21)
Neoplasms	56 (19)	Neoplasms	66 (18)
Musculoskeletal	28 (9)	Musculoskeletal	51(14)
Nervous	13 (8)	Metabolic	30 (8)
Digestive	19 (6)	Respiratory	24 (7)
Other	100 (33)	Other	117 (32)

Trials were classified as including a majority of older people if mean trial participant age ≥ 60 years

Medians, interquartile ranges (difference between 25 and 75^th^ percentile) and percentages are reported

For trials from both 2012 and 2019, the number of trials in the five most classified disease categories are shown

*Abbreviations*: *RCT* Randomized controlled trial, *IQR* Interquartile range

**Fig. 3:**
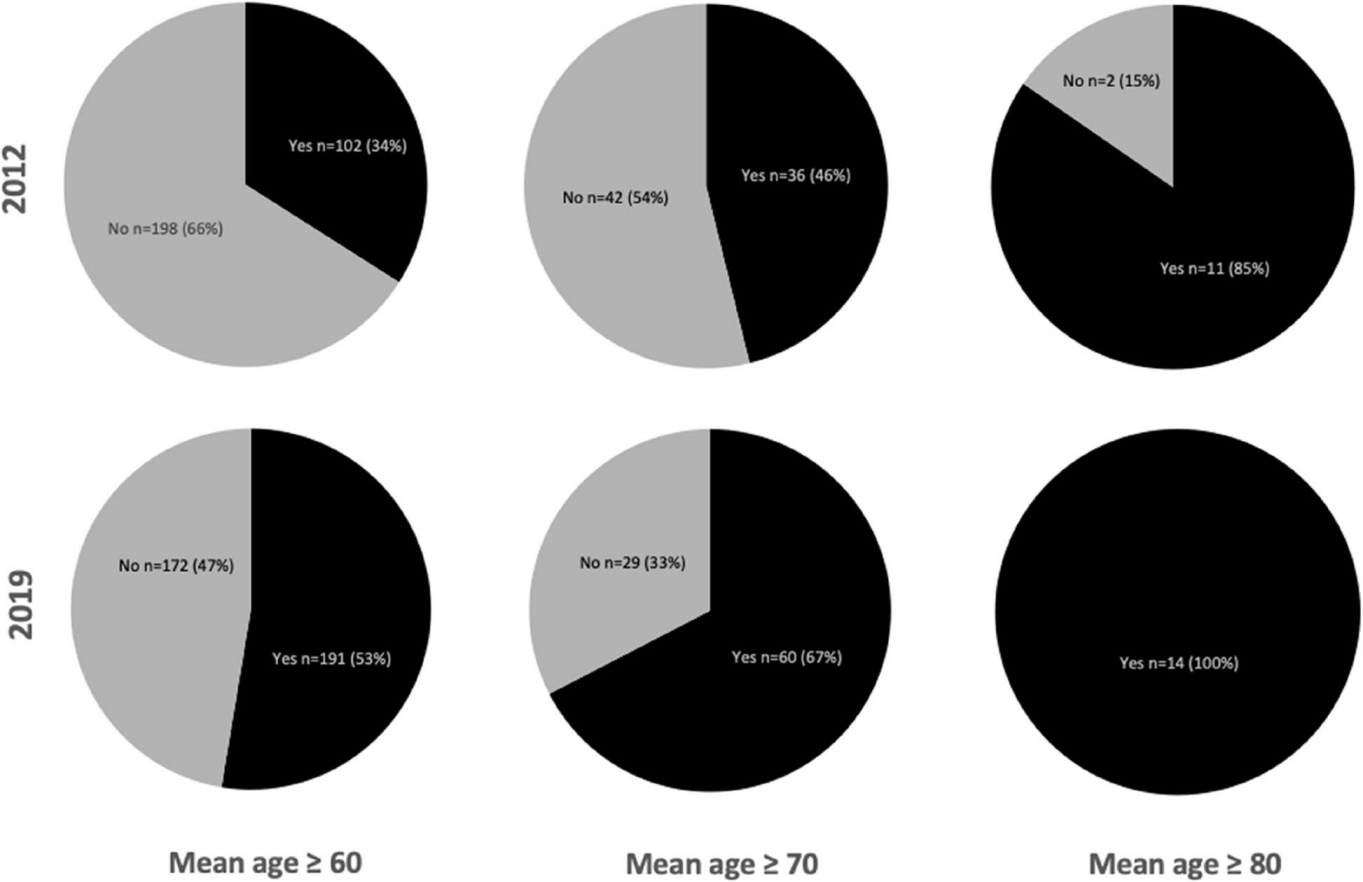
Proportion of RCTs including a majority of older people that report on geriatric assessments in patient characteristics in 2012 and 2019. The proportion of RCTs in 2012 and 2019 that describe geriatric assessments in either the population descriptives or the in- and exclusion criteria is shown for different mean age cutoffs. Abbreviations: RCT, randomized controlled trial

The proportion of RCTs including a majority of older people in 2019 and 2012 that describe geriatric assessments in the patient characteristics is shown in Fig. 3. In 2019, 191 of 363 (52%) trials including a majority of older people reported on somatic status, physical and mental functioning, social environment or frailty in the population descriptives or the in- and exclusion criteria. Of these 191 trials, these assessments were mentioned in the population descriptives in 158 studies, and in the in- and exclusion criteria in 115 studies. In 2019, 102 (53%) of 191 RCTs reported on more than one geriatric assessment compared to 34% in 2012. As sensitivity analysis, when trials with a mean age of 70 years and older were selected, 67% of trials reported on geriatric assessments in 2019 compared to 46% in 2012. When a mean age of 80 years and older was maintained, all trials (100%) described geriatric assessments in 2019 compared to 85% in 2012 (Fig. 3).

**Fig. 4:**
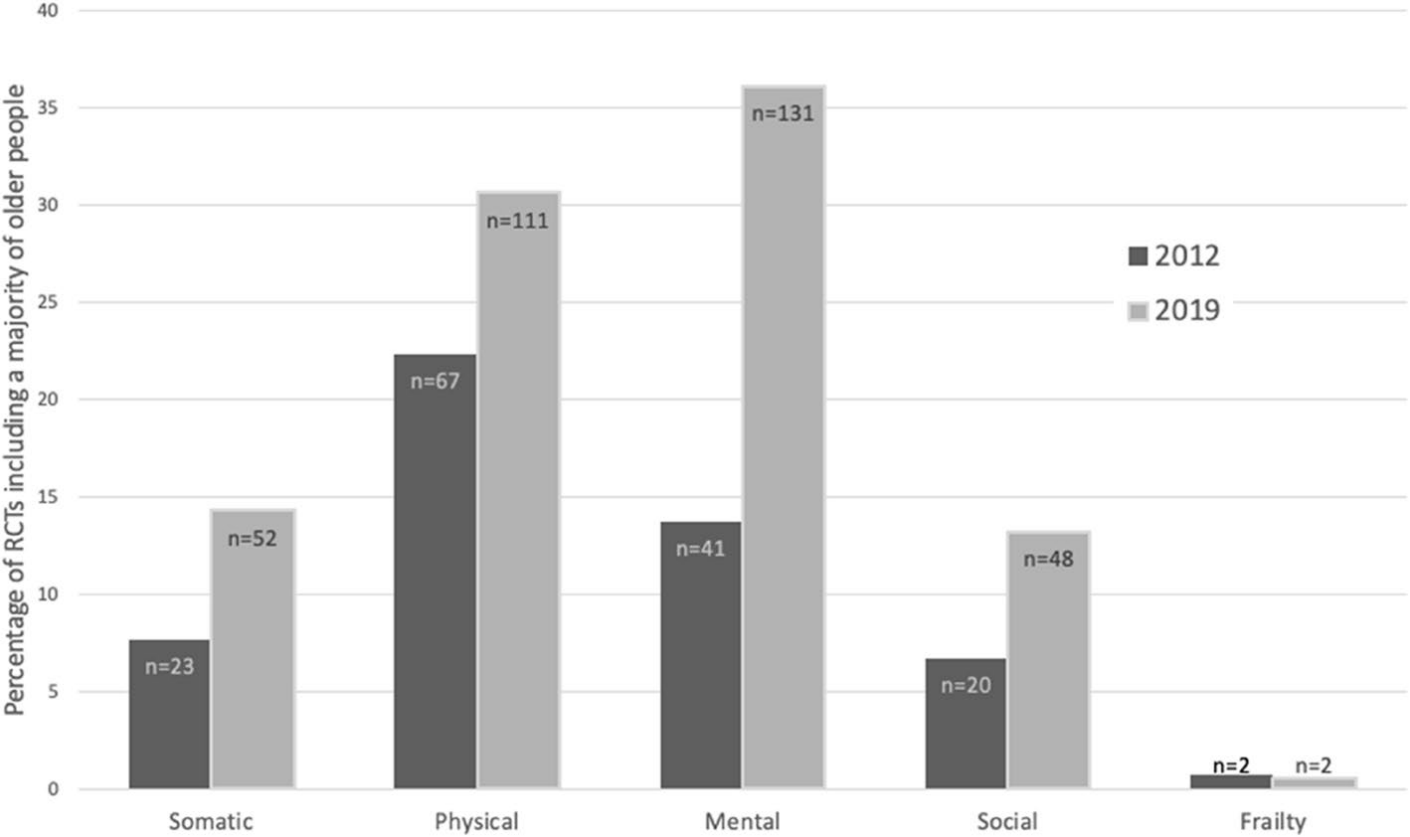
Proportion of RCTs including a majority of older people that report on geriatric assessments: somatic status, physical and mental functioning, social circumstances or frailty. RCTs including a majority of older people that report on geriatric assessments are shown as percentage of all RCTs including a majority of older people (mean participant age ≥ 60 years), divided by geriatric domain (somatic status, physical functioning, mental functioning, social circumstances and frailty). The total number of RCTs including a majority of older people was 300 in 2012 and 363 in 2019. Of all 2019 RCTs including a majority of older people, 102 (53%) trials reported on more than one geriatric domain. Abbreviations: RCT, randomized controlled trial

As shown in Fig. 4, in all RCTs including a majority of older adults in 2019, somatic status was described 52 times (14%), physical functioning 111 times (31%), mental functioning 131 times (36%), social circumstances 48 times (13%) and frailty 2 times (1%). Whereas in 2012 this was lower in all assessments: somatic status was described 23 times (8%), physical functioning 67 times (22%), mental functioning 41 times (14%), social circumstances 20 times (7%) and frailty 2 times (1%).

Of all RCTs including a majority of older adults in 2019, 87 trials (24%) had a primary outcome probably related to geriatric endpoints such as physical and cognitive functions. Since this data was not available for the reviews from the year 2012, no temporal trend could be assessed.

## Discussion

This review has 3 main findings. First, the proportion of trials specifically designed for older people did not increase between 2012 and 2019 (7% vs 8% resp). Second, the RCTs that included a majority of older people did increase from 21.9% in 2012 to 25.1% in 2019. Three, we found an increase in the percentage of RCTs including a majority of older people that reported on geriatric assessments (53% in 2019 vs 34% in 2012). All geriatric assessments were more reported in 2019, however this increase was most evident for the mental domain.

The proportion of trials specifically designed for older people did not increase between 2012 and 2019 (8% in 2019 vs 7% in 2012). Furthermore, no remarkable differences in median age, median participant number and median lower age cutoff value were found. These results are in line with the results of several other trials. For example, Ludmir et al. described increasing age disparities between participants in oncologic RCTs and oncologic disease populations [8]. Similarly, Ruiter et al. outline the underrepresentation of older people in approval documents in the Food and Drug Administration database [9]. Despite increasing calls for action and multiple recommendations to include more older individuals in clinical trials in the past years [10–16], these and our results suggest that older people are still underrepresented in medical research. However, a recently published study of Chow et al. describes some progress in the representation of older adults in practice-changing cancer trials, although they still remain underrepresented [17]. Together with the slight increase in trials that reported a majority of older people demonstrated in our review, this might point towards minor advances in the representation of older people in clinical trials and the external validity of these trials.

The higher percentage of trials reporting on geriatric assessments might be explained by the fact that the importance of the geriatric assessment has become increasingly acknowledged by both medical professionals and society over the past years. Several studies have shown that using geriatric assessment in RCTs improves decision making and thereby improving patient outcomes [18–20]. In particular, the number of clinical trials that report on the mental domain has remarkably increased over the past years. Since 2012, the significance of the mental domain for overall health has been more and more recognized and this, evidently, reflects in clinical research [21]. Another striking finding of our review is the persistently low use of specifically designed frailty indices or instruments. Although frailty is a good predictor of adverse outcomes such as falls, hospitalizations, morbidity and mortality, this domain is poorly represented in clinical trials. More attention is needed to define frailty in an objective manner. Therefore, a recently published review describes different frailty assessment considerations [22]. By reporting better on all these geriatric assessments in clinical trials, the results will be better translated to and applicable in clinical practice.

Several limitations may have influenced our results. Firstly, the random sample was only 10%, which can lead to a selection bias. Nonetheless, the results of this review are strikingly comparable with the results of the previous review of Broekhuizen et al., which implies a representative 10% sample [2]. Secondly, since the review is performed by different reviewers than previously, a certain subjectivity cannot be ruled out. Therefore, the possibility exists that the current reviewers have been more or less critical according to the geriatric assessment reporting. However, the exact same research methods were used in both the current and the previous reviews [2, 3]. This diminishes the probability of subjective trial assessment. Therefore, the results of both trials can easily be compared, and a clear conclusion can be drawn. Thirdly, no more than five years has passed between the current review and the previous reviews. This period of time might be too short for realizing improvements. Fortunately, after 2019 some promising articles were published about the implementation of clinical trials for older adults [23–26], which, hopefully, means the scientific attention for older people is already growing. Attempts to increase the inclusion of older people to randomized clinical trials should think of broaden the eligibility criteria and measure the more relevant endpoints to an elderly population. Lastly, we have used the expression RCTs specifically designed for older people for our first selection, however this expression might reflect that those studies took into account the complexity of older subjects which was not the case. However we used the same terminology as the previous reviews to enhance comparability”. 

## Conclusions

Within this systematic review we demonstrated that within 2019 the proportion of published RCTs designed for older people remains low, but that there is more attention for the description of the included older people by reporting more on geriatric assessments compared to 2012. However, we encourage RCTs to focus on the older population and their geriatric assessment in the patient characteristics even more to improve the external validity of these trials and to better connect to clinical practice.

## Supplementary Information

**Additional file 1.**

## Data Availability

Data used for the review is uploaded as supplementary material.

## Abbreviations

ICD-10: International Classification of Diseases 10
IQR: Interquartile Range
RCT: Randomized Controlled Trial
WHO: World Health Organization
